# Hepatitis C treatment outcomes among people who inject drugs experiencing unstable versus stable housing: Systematic review and meta-analysis

**DOI:** 10.1371/journal.pone.0302471

**Published:** 2024-04-26

**Authors:** Sarah Kimball, Marley Reynoso, Courtney McKnight, Don Des Jarlais

**Affiliations:** 1 Department of Epidemiology, New York University School of Global Public Health, New York, NY, United States of America; 2 Center for Drug Use and HIV/HCV Research, New York, NY, United States of America; Institute for Clinical and Experimental Medicine, CZECH REPUBLIC

## Abstract

**Background:**

The prevalence of hepatitis C virus (HCV) among people who inject drugs (PWID) is between 50–70%. Prior systematic reviews demonstrated that PWID have similar direct acting antiviral treatment outcomes compared to non-PWID; however, reviews have not examined treatment outcomes by housing status. Given the links between housing and health, identifying gaps in HCV treatment can guide future interventions.

**Methods:**

We conducted a systematic review using Preferred Reporting Items for Systematic Reviews and Meta-Analyses guidelines. We searched six databases for articles from 2014 onward. Two reviewers conducted title/abstract screenings, full-text review, and data extraction. We extracted effect measures for treatment initiation, adherence, completion, success, and reinfection by housing status. Studies underwent quality and certainty assessments, and we performed meta-analyses as appropriate.

**Results:**

Our search yielded 473 studies, eight of which met inclusion criteria. Only the treatment initiation outcome had sufficient measures for meta-analysis. Using a random-effects model, we found those with unstable housing had 0.40 (0.26, 0.62) times the odds of initiating treatment compared to those with stable housing. Other outcomes were not amenable for meta-analysis due to a limited number of studies or differing outcome definitions.

**Conclusions:**

Among PWID, unstable housing appears to be a barrier to HCV treatment initiation; however, the existing data is limited for treatment initiation and the other outcomes we examined. There is a need for more informative studies to better understand HCV treatment among those with unstable housing. Specifically, future studies should better define housing status beyond a binary, static measure to capture the nuances and complexity of housing and its subsequent impact on HCV treatment. Additionally, researchers should meaningfully consider whether the outcome(s) of interest are being accurately measured for individuals experiencing unstable housing.

## Introduction

While the prevalence of Hepatitis C virus (HCV) among the general adult population is 1% [1], among people who inject drugs (PWID) the prevalence is between 50–70% [2–4]. HCV is a blood borne virus that affects the liver and is commonly transmitted through sharing items to inject or prepare drugs [1]. While some people infected with HCV spontaneously clear the virus, over 50% go on to develop chronic HCV, which can result in serious and potentially fatal outcomes such as liver cancer [1]. Older interferon-based HCV treatments for chronic HCV had low efficacy and were difficult to complete due to side-effects and long courses of treatment [5]. In 2014, the advent of direct acting antiviral (DAA) medication revolutionized HCV treatment with highly effective treatments that were more tolerable and significantly shorter than older treatments [5]. DAAs expanded treatment options and access, particularly for populations that were previously considered harder to treat, including PWID [6].

In 2016, the World Health Organization (WHO) set a goal to eliminate hepatitis as a public health threat by 2030 [7]. Despite substantial progress, many of the target goals set for 2020 were unmet. Given the high prevalence of HCV, the WHO declared PWID as a priority population for HCV elimination efforts [8]. Among PWID, prior studies have found that those with unstable housing have a higher risk of acquiring HCV than their stably housed peers [9]. This aligns with prior research that has linked stable housing to positive mental and physical health outcomes; whereas unstable housing is often found to exacerbate existing issues and increase the risk of new ones [10–13]. These disparities could extend to HCV treatment outcomes; for example, unstable housing may result in individuals not only facing common barriers to treatment, such as cost [14,15], but also unique barriers such as the inability to safely and securely store medications [16]. We need to conduct research and design interventions to reach highly burdened populations, such as PWID who are experiencing unstable housing.

Prior systematic reviews have demonstrated that PWID have similar DAA treatment outcomes compared to non-PWID [17]; however, no reviews have looked at treatment outcomes among PWID who are unstably housed compared to those who are stably housed. Given the unique challenges of unstable housing, it is necessary to better understand current treatment outcomes to develop future interventions. To that end, this review seeks to answer the question: To what extent do HCV DAA treatment outcomes (initiation, adherence, completion, success, reinfection) differ between among PWID who are unstably versus stably housed?

## Methods

### Search strategy and selection

We used the Preferred Reporting Items for Systematic Reviews and Meta-Analyses (PRISMA) guidelines for our review [18]. The protocol was not registered, however the completed PRISMA checklists are available (S1 and S2 Checklists). The full search strategy is available in S1 Appendix. We used filters to limit results to studies published from 2014 onward. We searched Web of Science, Medline via PubMed, Embase, CINAHL Complete, PsychInfo, and Cochrane Library. All searches were completed on March 15, 2023. All search results were exported to EndNote 20 and de-duplicated. Results were then imported to Covidence and went through another check to de-duplicate. Covidence was used for title and abstract screening and full-text reviews. The screening and full-text reviews were completed by two reviewers (SK, MR). Any disagreements were discussed until a consensus was reached. After final articles were identified, we manually screened the articles’ references and performed a hand-search to identify any relevant studies that were not identified from the original search strategy. Additionally, relevant systematic reviews and meta-analyses were flagged during screening, and their references searched to identify additional studies.

We screened articles for the pre-specified inclusion and exclusion criteria. Studies were included if they collected quantitative data, included current or former PWID with chronic HCV, collected current housing information, used DAA treatment without interferon, tested outcomes in standard clinical settings, and were in English. HCV treatment outcomes needed to be stratified by housing status to answer our research question; therefore, some studies were excluded if they collected information about HCV but did not stratify treatment outcomes by housing status. Studies were excluded if they used interferon-based treatments as DAAs are now the most common treatment [19,20]. Studies were also excluded if they did not evaluate outcomes for an existing HCV treatment model. For example, studies of safety testing or evaluating a new intervention for HCV treatment were excluded as we sought to evaluate outcomes in existing care models. Studies occurring in prisons, jails, or in-patient hospital settings were excluded because institutional settings are different from community settings. Finally, systematic reviews and meta-analyses were excluded from the analysis.

### Data extraction and risk of bias

The primary outcomes of this review are HCV treatment initiation, adherence, completion, success, and reinfection. These outcomes reflect the steps of HCV treatment once the initial screening and linkage to care have been made [20–22]. Outcomes were only collected if the study reported on them and if they were stratified by housing status. For example, Seaman, et al. (2021) provided information for adherence and success; however, adherence was not disaggregated by housing status, so it was not included in our analysis [23]. Success, however, was disaggregated by housing status so that information was extracted and included in the results. For each outcome, we recorded the raw and adjusted effect measures, covariates controlled for, and sample size. Studies defined the adherence and success outcomes in different ways. For example, some studies defined success as sustained viral response (SVR) at 12 weeks (SVR12) whereas others defined it as SVR at 4 weeks [24–26]; therefore, for these outcomes we also extracted the specific definition each study used.

We also extracted information in the following categories: General (title, year of publication, journal, author(s), country and city/town study conducted in, funding source, conflict of interest), Methods (aims, study design, study start date, study end date, data sources, location of study), Participants (population description, total number of participants at baseline, total number of participants at end, inclusion criteria, exclusion criteria, recruitment method, baseline characteristics, definition of PWID, definition of housing status), Treatment Information (methods to diagnose HCV, treatment length, methods to assess reinfection, duration of follow-up, additional treatment information), Strengths, and Limitations.

Individual studies were assessed for risk of bias using the Newcastle-Ottawa Scale (NOS) for Quality Assessment for Cohort Studies and NIH Quality Assessment Tool for Observational Cohort and Cross-Sectional Studies (NIH), both of which rate studies using the categories of poor, fair, and good [27,28]. For cross-sectional studies, we only used the NIH tool as NOS is specific to cohort studies. Data extraction and quality checks were initially performed by one reviewer (SK) and checked by a second reviewer (MR). Any disagreements were discussed until consensus was reached.

### Data analysis

All outcomes were assessed using odds ratios. Reinfection was also assessed using incidence rate ratios. All effect measures assume the unstably housed group as the exposed group and stably housed as the unexposed group. Some studies reported stably housed as the exposed group; however, to keep comparisons consistent, we recalculated these effect measures so that unstably housed PWID were the exposed group. For example, Migdard, et al. (2021) reported that stably housed individuals had 1.67 times the odds of successful HCV treatment compared to unstably housed individuals [25]. We converted this odds ratio to reflect that unstably housed individuals had 0.60 times the odds of successful HCV treatment compared to stably housed individuals. One study reported housing status using five distinct categories, whereas all other studies reported it as a binary variable [29]. To allow for comparisons between studies, we collapsed the four categories of unstable housing into one “unstably housed” category for this study.

We performed a meta-analysis for treatment outcomes that had multiple measures that lent themselves to comparison via a meta-analysis. For this analysis, we used R version 4.11 using the *metabin* function and Mantel-Haenszel method. The significance was set at an alpha of 0.05. We anticipated studies having a high degree of heterogeneity given that we searched for studies in different countries and settings, for example national or citywide samples versus single clinics associated with syringe service programs (SSP) or needle and syringe programs; therefore, we used random-effects models for all meta-analyses. We used Paule-Mandel estimator to assess between-study variance and the Hartung-Knapp-Sidik-Jonkman adjustment for random effects models. We visually displayed results of the meta-analyses using forest plots. Finally, two people (SK, MR) assessed and came to consensus for the certainty of each outcome using Grading of Recommendations, Assessment, Development and Evaluations (GRADE) criteria, which provides four levels of certainty: very low, low, moderate, and high [30].

## Results

### Studies selected and characteristics

**Fig 1:**
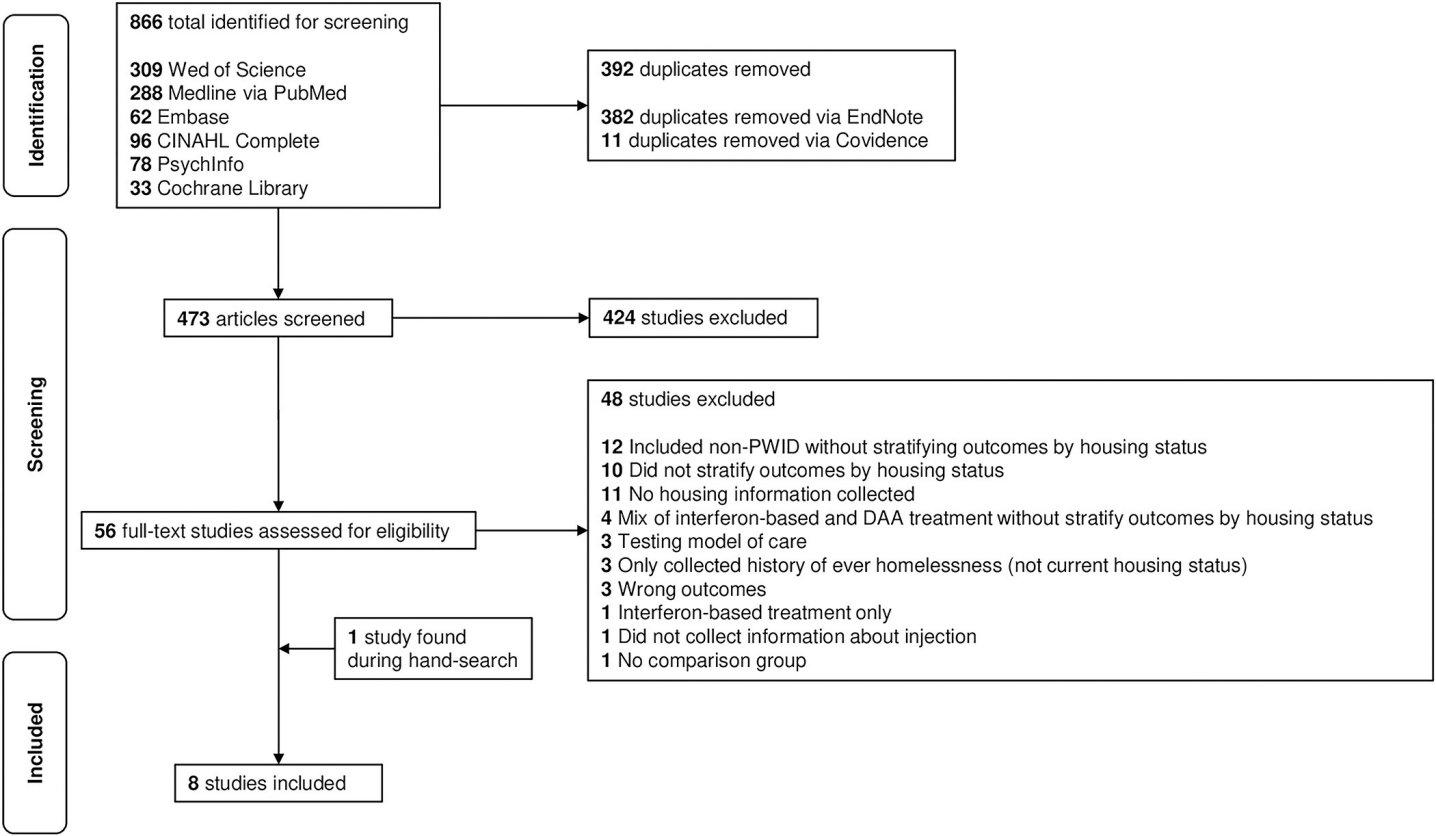
PRISMA flowchart.

Six databases were searched, resulting in 866 studies (Fig 1). After removing duplicates, we screened the titles and abstracts of 473 studies. The reviewers had 90.7% agreement initially and came to a consensus for all articles. We then performed full-text reviews of 56 studies which resulted in seven studies to include. The reviewers had 80.4% agreement initially and came to consensus for all articles. After manually screening the articles’ references and performing a hand-search, we identified one additional article to include. This resulted in eight total articles. The main reasons for excluding articles during the full-text review were either not collecting housing information or not stratifying HCV outcomes by housing status.

Several studies appeared to meet inclusion criteria but were ultimately excluded because they were testing a novel intervention to improve HCV treatment outcomes. For example, a study by Rosenthal, et al. (2020) examined whether concurrent prescribing of medications for opioid use disorder (MOUD) and HCV treatment improved treatment outcomes [31]. This study was ultimately excluded because it was testing a novel treatment model. While all included studies had participants using MOUD, it was not prescribed along with DAAs to evaluate changes in treatment outcomes. Since our study sought to compare outcomes of existing care models, new models to improve treatment outcomes were excluded. A study that ultimately met this existing care model criterion was Seaman, et al. (2021) which explored treatment outcomes among those on MOUD versus those in SSPs [23]. While some individuals were using MOUD, because it was evaluated in an existing care model using observational data, this study met inclusion criteria. We also excluded studies that only collected whether respondents had ever been homeless rather than current housing status because we were specifically interested in the impact of housing status on treatment outcomes.

All of the studies took place in urban environments except for one cross-sectional survey that was conducted in a national sample [32] (Table 1). Three studies were based in the United States [23,29,33], two in Australia [26,32], one in Canada [34], one in Norway [25], and one in the Czech Republic [35]. Since we wanted to explore natural treatment outcomes rather than, for example, a randomized-controlled trial to evaluate a new intervention, it is unsurprising that the included studies only consisted of cohort or cross-sectional designs. We ultimately included three cross-sectional studies [32–34], three retrospective cohort studies [26,29,35], and two prospective cohort studies [23,25]. The cohort studies all took place at medical clinics: three at low-threshold primary care clinics that focus on marginalized populations [23,25,26], one at a Hepato-Gastroenterology specialty clinic [35], and two at a clinic associated with a SSP [23,29].

**Table 1:** Study characteristics and risk of bias assessments.

Authors (Year)	Location	Study Timeframe	Study Design	Location of Study	Recruitment Method(s)	Data Source(s)	Study Outcome(s)	Risk of Bias: NOS	Risk of Bias: NIH
Butler, et al. (2019) [32]	Australia	June—August 2017	Cross-sectional	N/A	Snowball sampling; Convenience sampling (street outreach)	Survey (self-report)	Initiation	N/A	Poor
Corcorran, et al. (2021) [33]	Seattle, WA (USA)	June—November 2018	Cross-sectional	N/A	Respondent-Driven Sampling	Survey (self-report); HCV antibody testing	Initiation	N/A	Poor
Frankova, et al. (2021) [35]	Prague, Czech Republic	January 2017—August 2018	Retrospective cohort	Outpatient Specialty Clinic	N/A—chart review	Medical records	Adherence	Poor	Poor
Midgard, et al. (2021) [25]	Oslo, Norway	June 2013—June 2020	Prospective cohort	Primary Care Clinic	Convenience sampling (from clinic)	Medical records	SVR, Success	Good	Fair
Read, et al. (2017) [26]	Sydney, Australia	2015–2017	Retrospective cohort	Primary Care Clinic	N/A—chart review	Medical records	Success	Good	Fair
Seaman, et al. (2021) [23]	Portland, OR (USA)	May 2017—August 2018	Prospective cohort	Primary Care Clinic and Syringe Service Program	Convenience sampling (from clinic)	Medical records	Success	Poor	Poor
Socías, et al. (2019) [34]	Vancouver, Canada	April 2015—November 2017	Cross-sectional	N/A	Snowball sampling; Convenience sampling (street outreach)	Survey (self-report)	Initiation	N/A	Poor
Winetsky, et al. (2020) [29]	New York, NY (USA)	2015–2018	Retrospective cohort	Syringe Service Program	N/A—chart review	Medical records	Initiation, Completion, Success	Good	Fair

All of the studies had similar aims to explore HCV treatment outcomes and associated correlates impacting these outcomes. One study had a slightly different aim to see if there were differences in treatment outcomes between those at a MOUD clinic versus SSP; however, because information was collected about treatment outcomes, housing status, and met all other inclusion criteria, it was included in the analysis [23]. Many of the included studies had small sample sizes with two studies having under 100 participants [23,26], five studies having between 100 to 400 participants [25,29,32,33,35], and one study having 915 participants [34]. The main data sources were self-report surveys or medical records. Participants were primarily recruited through convenience [23,25,32,34], snowball [32,34], or respondent-driven sampling [33]. Three studies did not recruit participants because they retrospectively assessed existing medical records [26,29,35]. Since we wanted to evaluate treatment outcomes in standard clinical settings, we did not want incentives influencing outcomes. Some studies offered incentives to participate but importantly, these incentives were not tied to any treatment outcomes or exposure status [23,32,33]. For example, participants may have received an incentive to complete a baseline survey of sociodemographic information but did not receive an incentive for coming to treatment follow-up visits with their physician. One study evaluating reinfection rates offered incentives for follow-up visits, but this was done to prevent loss-to follow-up rather than impacting treatment outcomes themselves [25]. While any incentive in a study may impact other outcomes, we minimized this influence by not connecting them directly to outcomes or exposure of interest.

Five studies defined PWID as either current or former PWID [23,25,26,34,35]. Only two studies limited their population to current PWID, defined as either having injected in the past six or twelve months (Table 2) [32,33]. One study did not state if they included current and former PWID or just current PWID, but the study did take place at a SSP indicating that there was likely a high proportion of current PWID [29]. Housing status categories and definitions varied between studies. Some studies used the term “homeless” or “houseless” while others used “unstable housing.” Only three studies provided clear definitions of these terms. One study defined unstable housing as homelessness, rough sleeping, hostels, refuges, or couch surfing [32]. Another study defined unstable housing as low-threshold or temporary accommodations [25]. The third study defined unstable housing through different categories of street homeless, shelter, single room occupancy hotel, or staying with friends or family [29]. This was the only study that stratified treatment outcomes by multiple categories of housing instability; however, since all other studies reported anyone meeting their definition of homeless or unstably housed into one category, we collapsed these categories for our data analysis. The stable housing categories also had few definitions. Three studies defined this as “not homeless” [26,33,34], while four studies defined this as simply “stable housing” [23,29,32,35]. Only one study provided a definition of stable housing as owning or renting a house or apartment [25].

**Table 2:** PWID, unstable housing, and stable housing definitions and HCV diagnosis and treatment characteristics by study.

Authors	PWID Definition	Unstable Housing Categories and Definitions	Stable Housing Categories and Definitions	Diagnosis Method	Treatment Lengths	Additional Support
Butler, et al. (2019) [32]	Current PWID (IDU in past 6 months)	Unstable housing: homelessness, rough sleeping, hostels, refuges, couch surfing	Stable housing	Self-report	Did not specify	N/A
Corcorran, et al. (2021) [33]	Current PWID (IDU in past 12 months)	Currently homeless	Currently not homeless	Rapid HCV antibody test and self-report	Did not specify	N/A
Frankova, et al. (2021) [35]	Current and former PWID	Unstable housing	Stable housing	HCV RNA test	8 or 12 weeks	None specified
Midgard, et al. (2021) [25]	Current and former PWID	Unstable housing: low-threshold accommodation, institution, or prison	Stable housing: owning or renting housing/apartment	HCV RNA test	Did not specify	Medication support options
Read, et al. (2017) [26]	Current and former PWID	Homeless in last 12 months	Not homeless in last 12 months	HCV RNA test	8, 12, or 24 weeks	Standard or enhanced medication support options
Seaman, et al. (2021) [23]	Current and former PWID	Houseless/unstable	Stable/transitional	HCV RNA test	12 weeks	None specified
Socías, et al. (2019) [34]	Current and former PWID	Homelessness in last 6 months	No homelessness in last 6 months	Self-report	Did not specify	N/A
Winetsky, et al. (2020) [29]	Did not specify if included both current and former	Four categories: street homeless, shelter, single room occupancy hotel, staying with friends or family	Stably housed	HCV RNA test	Did not specify	On-site medication adherence support with case manager

The confirmatory testing for current HCV infection varied across studies. Five studies used HCV RNA testing [23,25,26,29,35], and one used rapid HCV antibody tests to confirm presence of HCV with self-report questions to confirm if they were ever successfully cured [33]. Two studies relied on self-report for being diagnosed with HCV and never having been successfully cured [32,34]. Treatment lasted between 8–24 weeks depending on the study [23,26,35], although five studies did not specifically report treatment length [25,29,32–34]. Finally, among the five cohort studies, three specified that their offices provided additional support for medication adherence [25,26,29], while the other two did not specify if there was or was not added support [23,35].

For our specific research question, the studies generally received quality ratings of “poor” or “fair” using the NOS and NIH assessments. Many studies did not provide clear definitions of housing status [23,26,33–35], and if they did [25,32], all but one presented housing as a binary variable of homeless or unstably housed versus not, rather than disaggregating housing into different categories such as street homeless, shelter homeless, or single-room occupancy hotels [29]. Several studies also relied on self-report data for diagnosis of HCV and treatment outcomes [32–34], rather than medical records or RNA tests [23,25,26,29,35]. Finally, several studies had small sample sizes [23,26,29,35], and three studies were cross-sectional, thereby limiting our analysis of temporality [32–34].

### Initiation

Four studies measured HCV treatment initiation: three cross-sectional studies and one retrospective cohort study [29,32–34]. All studies found a lower odds of treatment initiation in the unstably housed group, although only three studies were significant (Tables 3 and S1). Three of the four studies provide adjusted odds ratios, two of which remained significant after adjustment (aOR = 0.42; 95% confidence interval (CI) = 0.22, 0.82; adjusted for current MOUD and unemployment) [32]; (aOR = 0.39; 95% CI = 0.19, 0.80; adjusted for age and gender) [33]. One of the studies was no longer significant after adjusting for age, gender, race, HIV status, heroin injection frequency, crack use frequency, high-risk drinking, HCV care engagement, residency, employment, income, and incarceration history (aOR = 0.74; 95% CI = 0.28, 1.69) [34].

**Table 3:** Study outcomes and GRADE certainty assessments.

**Study**	**Initiation**		**Adherence**		**Completion**		**Success**		**Reinfection**	
	**N**	**OR** ^**a**^	**N**	**OR**	**N**	**OR**	**N**	**OR**	**N**	**OR**
		**(95% CI),**		**(95% CI),**		**(95% CI),**		**(95% CI),**		**(95% CI),**
		**p-value**		**p-value**		**p-value**		**p-value**		**p-value**
Butler, et al. (2019) [32]	289	0.38	-	-	-	-	-	-	-	-
		(0.20–0.73),								
		p = 0.003*								
Corcorran, et al. (2021) [33]	244	0.31	-	-	-	-	-	-	-	-
		(0.16–0.60),								
		p = 0.005*								
Frankova, et al. (2021) [35]	-	-	101	0.1	-	-	-	-	-	-
				(0.01, 0.48),						
				p < 0.001*						
Midgard, et al. (2021) [25]	-	-	-	-	-	-	323	0.6	267	0.94
								(0.22–1.66),		(0.23, 3.83),
								p = 0.323		p = 0.931
Read, et al. (2017) [26]	-	-	-	-	-	-	67	0.27	-	-
								(0.08–0.95),		
								p = 0.042		
Seaman, et al. (2021) [23]	-	-	-	-	-	-	50	3.93	-	-
								(0.45–34.43),		
		p = 0.36								
Socías, et al. (2019) [34]	915	0.43	-	-	-	-	-	-	-	-
		(0.22–0.76),								
		p = 0.007*								
Winetsky, et al. (2020) [29]	102	0.65	-	-	58	1.62	48	0.58	-	-
		(0.27–1.60),				(0.40, 6.63),		(0.059–5.674),		
		p = 0.353		p = 0.503		p = 0.637			
GRADE	Low		Very Low		Very Low		Very Low		Very Low	

*p < 0.05.

^a^Odds Ratio.

**Fig 2:**
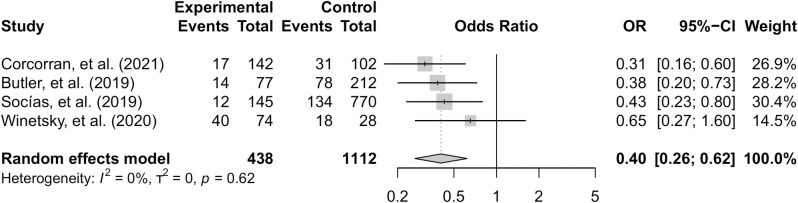
Treatment initiation forest plot.

The four studies used consistent definitions of treatment initiation and were comparable enough for a meta-analysis. Using a random-effects model, the pooled odds ratio indicates that those with unstable housing had 0.40 (95% CI = 0.26, 0.62) times the odds of treatment initiation compared to those with stable housing (Fig 2). The I^2^ was 0% indicating minimal heterogeneity; however, the I^2^ value was not precise (95% CI = 0.0%, 84.7%) and given the small number of studies, I^2^ may be biased downward [36]. Additionally, studies were from different countries, used different recruitment methods, and different definitions of PWID and housing status; therefore, despite a low I^2^, we used a random-effects model. While three of the studies provided adjusted odds ratios, these were not included in the meta-analysis as they adjusted for different covariates.

### Adherence

Only one study measured adherence, which used a retrospective cohort design to evaluate treatment outcomes using medical records at a Hepato-Gastroenterology clinic in Prague, Czech Republic. They found that those with unstable housing had 0.10 (95% CI = 0.02, 0.48) times the odds of adhering to their treatment compared to those with stable housing, however, adherence was not clearly defined [35]. Although they specified that the measure included missed appointments and missed or delayed doses dispensed, they did not specify when someone would be categorized as non-adherent. For example, if someone missed one appointment, it was unclear if that made them non-adherent or if they needed to miss more appointments to be considered non-adherent. Additionally, missed appointments may not truly correlate with one’s adherence to medication. For example, the authors themselves note their finding of a stable job negatively impacting adherence may be because it is harder to regularly attend appointments due to work conflicts, rather than not taking their medications consistently [35]. Interestingly, regardless of adherence status, 98.02% of participants achieved SVR12 using an intent-to-treat (ITT) analysis [35]. Unfortunately, these outcomes were not disaggregated by housing status and therefore, were not included in the success outcome analysis. The study also identified older age (OR = 1.07; 95% CI = 1.0, 1.20) as positively impacting adherence and stable jobs as negatively impacting adherence (OR = 0.24; 95% CI = 0.06, 0.8) [35].

### Completion

One study measured treatment completion, using a retrospective cohort design to analyze medical records from a clinic associated with an SSP in New York City. They found that those with unstable housing had 1.62 (95% CI = 0.40, 6.63) times the odds of treatment completion compared to those who were stably housed [29]. This finding is not significant and has a large confidence interval due to the small sample size (n = 58), with the stably housed group only having 18 participants [29]. While the study collected housing status using five distinct categories (street homeless, shelter, staying with friends or family, single-room occupancy hotel, stably housed), we collapsed the four unstable housing statuses into one category due to small sample sizes and to aid in comparison with other studies in this review. While the study used multivariable logistic regression, housing status was not included because it was not found to be significant at α = 0.10 during backwards elimination [29]. Interestingly, this study assessed housing status at three separate points of treatment: intake, during treatment, and at the end of treatment, although all analyses used housing status at intake. They found that five participants who were street homeless at intake transitioned to some type of housing over the course of treatment, while one person in a shelter became street homeless [29]. However, the small sample size and inability to compare these transitions to those who were lost-to-follow-up prevent further analysis.

### Success

Four studies provided success measurements: two prospective cohort studies and two retrospective cohort studies. Two studies took place within integrated primary care settings focused on serving PWID and other vulnerable populations [25,26]. Two studies were in clinics co-located with SSP and/or MOUD programs [23,29]. All four study clinics provided social services and/or harm reduction programs. Despite similar settings, the measures themselves had inconsistent definitions and analyses that made them incompatible for meta-analysis (Table 4).

**Table 4:** HCV treatment success definitions and outcomes.

Study	Success Definition	ITT^a^			Modified			SVR Among those Tested	
		Definition	N	OR^b^ (95% CI), p-value^c^	Definition	N	OR (95% CI), p-value	N	OR (95% CI), p-value
Midgard, et al. (2021)	SVR at 4 or more weeks or end of treatment SVR if no additional result available	All those that started treatment	340	90%^c^	**mITT**^**d**^ Dropped those lost to follow-up during treatment	323	0.60 (0.22, 1.66), p = 0.32	311	98.39%^c^
Read, et al. (2017) [26]	SVR at 12 weeks	All those that started treatment	72^e^	0.2718 (0.08, 0.96), p = 0.04*	**mITT**^**d**^ Dropped those with undetectable HCV RNA at end of treatment but no SVR test at 12 weeks and those who experienced treatment interpretation	65	92%^c^	59	100%^c^
Seaman, et al. (2021) [23]	SVR at 12 weeks	All those that started treatment	50	3.93 (0.45, 34.43), p = 0.36	**mPP**^**f**^ Dropped those lost to follow-up during treatment	41	95.12%^c^	41	95.12%^c^
Winetsky, et al. (2020) [29]	SVR (did not provide timeframe)	All those that started treatment	58	1.04 (0.44, 2.44), p = 0.93	Study did not provide definition^g^	48	0.58 (0.06, 5.67), p = 0.64	-	-

* p< 0.05.

^a^Intent-to-Treat.

^b^Odds Ratio.

^c^If success was not broken down by housing status the measure provided represents the percentage of success for entire sample.

^d^Modified Intent-to-Treat.

^e^Only 67 used for housing status analysis because 5 did not provide housing data.

^f^Modified Per Protocol.

^g^OR in table calculated by excluding those that did not complete treatment.

Two studies used SVR12 to determine treatment success [23,26]. One study used either SVR at four or more weeks or SVR at the end of treatment to define success [25]. One study defined success as SVR but did not provide information about when the SVR test was completed [29]. Furthermore, not all analyses were stratified by housing status. Two studies conducted ITT analyses as well as modified intent-to-treat (mITT), but only provided housing status information for the ITT [23,26]. Conversely, one study only provided housing status for the mITT but not ITT analysis [25]. The fourth study did not explicitly conduct ITT or mITT analyses but provided the raw data which enabled us to conduct an ITT analysis using all individuals who started treatment as the denominator [29]. While they also did not conduct a mITT, we calculated it using the ITT sample, but excluded those that did not complete treatment. Due to these inconsistencies across studies, it was inappropriate to run a meta-analysis using all four effect measures.

While we were unable to run a meta-analysis for this outcome, we did evaluate the individual study outcomes. Three of the studies found lower odds of treatment success for those with unstable housing compared to stable housing [25,26,29], although only one result was significant (OR = 0.27; 95% CI = 0.08, 0.95) [26]. One study found that those with unstable housing had higher odds of treatment success (OR = 3.93; 95% CI = 0.45, 34.43), but this finding was not significant and had a wide confidence interval due to the small sample size (n = 50) [23]. Three out of the four studies also reported the proportion of successful SVR tests among only those that returned for it. For all three of those studies, the success rate for SVR was high among those that were tested (95.12%; 98.39%; 100%) [24–26], but unfortunately, no studies disaggregated these results by housing status so we could not compare the results based on housing. Both Read et al. (2017) and Seaman et al. (2021) note that these high success rates among those that came back for the test may indicate that the lower ITT rates reflect high loss-to-follow-up rather than true treatment success [23,26].

### Reinfection

One study measured reinfection using a prospective cohort design. The study followed those who achieved SVR in a low threshold clinic co-located with harm reduction services in Oslo, Norway. They defined reinfection as HCV RNA recurrence following SVR with the individual having a higher risk of reinfection over relapse [25]. They used this distinction because they did not perform additional viral sequencing, so they could not confirm biologically whether it was a case of reinfection or relapse [25]. They found that those who were unstably housed had 0.94 (95% CI = 0.23, 3.83) times the odds of reinfection compared to those who were stably housed [25]. However, there was low occurrence of reinfection, with eight people in total reinfected, four in each housing category; this resulted in a wide confidence interval [25]. Additionally, the follow-up time varied between the two groups, so the incidence rate may be a more appropriate measure. Among those with unstable housing the incidence rate was 3.47 (0.95–8.89) per 100 person-years and among those with stable housing it was 2.07 (0.56–5.31) per 100 person-years [25].

Out of the eight individuals who were reinfected, all were actively injecting and all were successfully retreated [25]. Reinfection was also associated with younger age, unemployment, and lower education level [25]. For the entire sample, the median follow-up was 0.50 years [25]. This short follow-up duration may explain the low incidence of reinfection, although five out of the eight reinfections occurred within the first year of follow-up, indicating that reinfection may happen early after initial SVR [25].

### Certainty assessment

Treatment initiation had “low” certainty using the GRADE criteria. Despite the consistent findings and significant pooled odds ratio, there were only four studies that measured this outcome and there was variable quality across the studies. The remaining outcomes of treatment adherence, competition, success, and reinfection all had “very low” certainty using the GRADE criteria. For adherence, completion, and reinfection, there was only one study each, severely limiting the certainty of the findings. While the success outcome included four studies, they used varying definitions of success and different analyses, making them incompatible for a meta-analysis.

### Reporting bias

Due to limited measurements, we could not perform statistical tests such as funnel plots to check for reporting bias. To reduce reporting bias, we reviewed citations of included articles and systematic reviews and meta-analyses found during screening to identify additional relevant studies.

## Discussion

Consistent with prior studies about housing and health, unstable housing generally led to poorer HCV treatment outcomes among PWID; however, given the limited number of studies, further research is needed to better understand gaps in HCV treatment. Initiation was the only outcome with multiple studies amendable to a meta-analysis, and results indicate that treatment initiation is significantly lower among those with unstable housing compared to their stably housed peers. Previous studies have identified barriers between screening positive for HCV and beginning treatment. For example, before starting treatment patients often undergo testing such as additional testing to determine viral genome and liver fibrosis [23]. These additional tests can be laborious and prior studies have found success in bundling tests into one visit rather than multiple visits [23]. Other studies have found challenges with health insurance and paying for treatment, particularly in settings like the United States that lack universal healthcare [37]. Although interestingly, in our review treatment initiation gaps persisted even in countries that had universal healthcare, such as Canada and Australia, indicating the presence of additional barriers [32,34]. PWID, particularly those with unstable housing, may also face unique barriers to treatment initiation. For example, despite prior studies demonstrating that treatment outcomes are comparable between PWID versus non-PWID [17], providers are hesitant to provide treatment due to concerns about poor adherence or reinfection [33,35,38]. This stigma is likely exacerbated among PWID without stable housing. Conversely, individuals may be hesitant to initially go to providers due to stigmatizing past experiences, indicating a need for trauma-informed, non-stigmatizing medical practices [39]. Future studies should explore barriers to treatment initiation, especially barriers that may be unique to PWID without stable housing.

Despite identifying relevant findings for treatment initiation, overall, this review identified few studies focused on HCV treatment and housing status, and among those included, most did not specifically focus on housing but rather included it as one of many variables. While it may be beneficial to explore the association between different covariates and HCV treatment outcomes, housing is a complex variable and given the high prevalence of HCV among PWID and the unique challenges for those experiencing unstable housing, studies specifically focusing on this population are needed. For example, there are many different and distinct forms of housing instability such as street homelessness, shelter homelessness, or single-room occupancy hotels [40]. All but one study collected housing information as a binary variable and in the one study that did collect multiple housing statuses, we had to collapse several categories into one due to small sample sizes and to enhance comparability with other studies. While different housing categories may fall under the broad category of unstable, they are unique environments that can plausibly impact HCV treatment in different ways. For example, storing medications may be a bigger barrier for someone experiencing street homelessness than someone in a shelter. Understanding similarities and differences in treatment based on these specific housing statuses can help design and tailor future interventions. Additionally, housing status is not a static variable and can change over time [40]; however, all but one study collected housing status at only one point in time rather than as a time-varying covariate. For the one study that collected it at multiple points, they only included housing status at intake in their analyses despite noting that housing changed for some participants during treatment [29]. Future studies should be longitudinal as housing is a dynamic variable that can change over the course of treatment [29]. Changes in housing status can be destabilizing and impact HCV treatment outcomes in ways that consistent housing statuses may not.

Future studies should also consider how they define outcomes and how these definitions may not accurately measure what is occurring for people experiencing unstable housing. For example, in the study evaluating treatment adherence, part of the measure incorporated attendance at medical visits [35]. Individuals with unstable housing often have competing needs that may take priority over appointments, such as securing food or money. Additionally, missed appointments do not necessarily correlate to missed medication doses, which would be a more accurate measure of adherence. Similarly, many of the success measures required blood tests weeks after treatment was finished. Given that the efficacy of many DAAs is over 90%, it is not unreasonable that someone would choose not to return for a confirmation blood test after completing treatment, particularly when they have other pressing needs [14]. This is further supported by the high treatment efficacy among those who returned for SVR testing; one study went as far as to conclude that by using an ITT analysis with SVR12 as the outcome, they were effectively measuring loss to follow-up rather than true SVR rates [26]. We should consider using alternative approaches to measuring treatment success, particularly for those with unstable housing, such as end of treatment SVR rather than SVR12. While this may stray from traditional SVR12 measures, it can improve our understanding of what is truly occurring, which is necessary to identify gaps and solutions. For example, an intervention to improve treatment completion may look different from an intervention to improve SVR12 follow-up rates.

There are several other limitations to consider. All but two studies grouped both former and current PWID together into one category. Given that HCV is commonly spread through shared injection equipment, there may be important differences between former PWID and those that continue to inject [1]. Additionally, injection status could change over time. Therefore, future studies should consider stratifying outcomes based on injection status or measuring injection status as a time-varying covariate. Finally, many of the study samples were small resulting in wide confidence intervals.

There are limitations to our review. We only included published studies, which may have resulted in publication bias. We also focused exclusively on HCV treatment rather than looking at other outcomes in the HCV testing and treatment cascade such as antibody testing or RNA testing. Finally, we excluded studies if they met inclusion criteria except for stratifying treatment outcomes by housing status. These studies likely collected all relevant information, but we could not include them as they were not broken down by housing status for our specific outcomes of interest.

In order to reach HCV elimination targets, we must reach highly burdened populations such as PWID experiencing unstable housing. As previous studies have found, lack of safe and secure housing may negatively impact HCV treatment outcomes, although our conclusions are limited by lack of evidence. Future studies should more precisely measure housing status beyond binary, static measures, as well as consider modifying how outcomes are defined to better capture the outcome of interest. These improvements will result in informative studies to address the current lack of evidence and better elucidate the causal pathways between housing and HCV treatment outcomes. Given there is now effective and tolerable DAA treatment, further research is urgently needed to identify key areas to intervene and cure populations highly burdened by HCV.

## Supporting information

S1 Checklist PRISMA checklist. (DOCX)

S2 Checklist PRISMA abstract checklist. (DOCX)

S1 Appendix Search strategy. (DOCX)

S1 Table Raw data for outcome odds ratios. (DOCX)
