# Supplementary material for: Hepatitis C treatment outcomes among people who inject drugs experiencing unstable versus stable housing: Systematic review and meta-analysis
Source: PLoS One. 2024 Apr 26;19(4):e0302471. doi: 10.1371/journal.pone.0302471 (PMC11051606; doi:10.1371/journal.pone.0302471)
Supplement: S1 Appendix — (DOCX) [file pone.0302471.s003.docx]

**S1 Appendix. Search strategy**

(“hepatitis C” OR HCV)

AND

(treatment OR therapeutic OR therapy OR medication OR care OR healthcare OR modality OR DAA OR “direct acting antiviral” OR therapy OR intervention OR pharmaceutical OR antiviral OR “antiviral agent” OR Harvoni OR Zepatier OR Epclusa OR Vosevi OR Mavyret OR Sovaldi OR Daklinza OR Viekira Pak OR Exviera OR Simeprevir)

AND

(“people who inject drugs” PWID OR “intravenous drug use” OR IDU OR IVDU OR intravenous)

AND

(homeless OR homelessness OR shelter OR housing instability OR unsheltered OR unhoused OR houseless OR “displaced” OR Medicaid OR housing OR home OR “housing status” OR permanent)

| **Database** | **Filter** |
| --- | --- |
| Web of Science | 2014-2023 (selected on checklist) |
| Medline via PubMed | 2014-2023 |
| Embase | 2014-current |
| CINAHL Complete | 2014-2023 |
| PsychInfo | 2014-2023 |
| Cochrane Library | 1/1/2014 to 3/15/2023 |
