# Supplementary material for: Hepatitis C treatment outcomes among people who inject drugs experiencing unstable versus stable housing: Systematic review and meta-analysis
Source: PLoS One. 2024 Apr 26;19(4):e0302471. doi: 10.1371/journal.pone.0302471 (PMC11051606; doi:10.1371/journal.pone.0302471)
Supplement: S1 Table — (DOCX) [file pone.0302471.s004.docx]

**S1 Table. Raw data for outcome odds ratios**

| **Study** | **Initiation** | | | **Adherence** | **Completion** | | | **Success** | | | **Reinfection** | | |  |
| --- | --- | --- | --- | --- | --- | --- | --- | --- | --- | --- | --- | --- | --- | --- |
|  |  |  |  |  |  |  |  |  |  |  |  |  |  |  |
| Butler, et al. (2019) |  | **UH^a^** | **SH^b^** | - | - | | | - | | | - | | |  |
|  | **Initiation** | 14 | 78 |  |  |  |  |  |  |  |  |  |  |  |
|  | **Non-Initiation** | 63 | 134 |  |  |  |  |  |  |  |  |  |  |  |
| Corcorran, et al. (2021) |  | **UH** | **SH** | - | - | | | - | | | - | | |  |
|  | **Initiation** | 17 | 31 |  |  |  |  |  |  |  |  |  |  |  |
|  | **Non-Initiation** | 125 | 71 |  |  |  |  |  |  |  |  |  |  |  |
| Frankova, et al. (2021) | - | | | Did not report raw data | - | | | - | | | - | | |  |
| Midgard, et al. (2021) | - | | | - | - | | |  | **UH** | **SH** |  | **UH** | **SH** |  |
|  |  |  |  |  |  |  |  | **Success** | 160 | 146 | **Reinfection** | 4 | 4 |  |
|  |  |  |  |  |  |  |  | **Non-Success** | 11 | 6 | **Non-Reinfection** | 149 | 140 |  |
| Read, et al. (2017) | - | | | - | - | | |  | **UH** | **SH** | - | | |  |
|  |  |  |  |  |  |  |  | **Success** | 13 | 41 |  |  |  |  |
|  |  |  |  |  |  |  |  | **Non-Success** | 7 | 6 |  |  |  |  |
| Seaman, et al. (2021) | - | | | - | - | | |  | **UH** | **SH** | - | | |  |
|  |  |  |  |  |  |  |  | **Success** | 11 | 28 |  |  |  |  |
|  |  |  |  |  |  |  |  | **Non-Success** | 1 | 10 |  |  |  |  |
| Socías, et al. (2019) |  | **UH** | **SH** | - | - | | | - | | | - | | |  |
|  | **Initiation** | 12 | 134 |  |  |  |  |  |  |  |  |  |  |  |
|  | **Non-Initiation** | 133 | 636 |  |  |  |  |  |  |  |  |  |  |  |
| Winetsky, et al. (2020) |  | **UH** | **SH** | - |  | **UH** | **SH** |  | **UH** | **SH** | - | | |  |
|  | **Initiation** | 40 | 18 |  | **Completion** | 34 | 6 | **Success** | 30 | 4 |  |  |  |  |
|  | **Non-Initiation** | 34 | 10 |  | **Non-Completion** | 14 | 4 | **Non-Success** | 13 | 1 |  |  |  |  |

^a^Unstable Housing

bStable Housing
